# CcpA-mediated regulation of cellular energy metabolism in the ruminal bacterium *Streptococcus bovis*

**DOI:** 10.1128/spectrum.02150-24

**Published:** 2025-06-25

**Authors:** Yunan Weng, Yaqian Jin, Wenze Zhang, Lihong Wang, Hongrong Wang

**Affiliations:** 1 Laboratory of Nutrition and Metabolism of Herbivorous Animals, College of Animal Science and Technology, Yangzhou University 614678 , Yangzhou, Jiangsu, China; 2 Institute of Animal Nutrition and Metabolism Regulation, College of Animal Science and Technology, Yangzhou University 614678 , Yangzhou, Jiangsu, China; 3 College of Agriculture and Biology of Liaocheng University, Liaocheng University 697434, Liaocheng, Shandong, China; Fujian Agriculture and Forestry University, Fuzhou City, Fujian, China

**Keywords:** energy metabolism, catabolite control protein A, *ccpA*, *Streptococcus bovis* S1, KEGG pathway, random forest

## Abstract

This study investigated the cellular energy metabolite profiles of *Streptococcus bovis* S1. Glucose concentrations of 5 or 50 mM and the presence or absence of *ccpA* were applied during the cultivation of *S. bovis* S1. Results revealed the identification of 51 metabolites categorized into seven types: amino acid derivatives, amino acids, coenzymes and vitamins, nucleotides and their metabolites, organic acids and their derivatives, phosphate sugars, and phosphoric acids. Each group exhibited distinct cellular energy metabolite profiles, as evidenced by principal component analysis showing unique metabolite compositions. Orthogonal projections to latent structures discriminant analysis (OPLS-DA) further distinguished between each pair of groups. KEGG prediction indicated that the abundance and enrichment of metabolites were primarily involved in carbon metabolism, amino acid metabolism, and nucleotide metabolomics. Based on the random forest algorithm, glutamine and UDP-GlcNac were identified as biomarkers under varying glucose conditions and in the presence or absence of *ccpA*. Further analysis reveals that low glucose restricts carbon flux to the Embden-Meyerhof-Parnas (EMP) pathway, while *ccpA* knockout further reduces flux through this pathway. Glucose and CcpA might regulate fructose-1,6-bisphosphate (FBP) concentration to modulate lactate dehydrogenase and pyruvate formate-lyase enzyme activity, thereby influencing the fermentation direction of pyruvate. In low-glucose environments, glutamine serves to alleviate glucose deficiency, and the interaction between glucose and CcpA may mediate the fate of amino acids differently. Low glucose limits guanine synthesis but not adenine, cytosine, or thymine synthesis, while *ccpA* knockout disrupts both synthesis pathways by inhibiting the pentose phosphate pathway (PPP). Overall, this study provides insights into the intricate interplay between glucose concentration, *ccpA* knockout, and cellular energy metabolism in *Streptococcus bovis* S1.

**IMPORTANCE:**

*S. bovis* S1 plays a pivotal role in lactate production in the rumen, increasing the risk of rumen acidosis. Modulating the fermentation profile of *S. bovis* S1 could reduce lactate accumulation, potentially improving rumen health. In this study, *ccpA* knockout decreased fermentation fluid lactate concentration but increased formate concentration. Liquid chromatography tandem mass spectrometry characterized the metabolic activity of *S. bovis* S1 under varying glucose concentrations. We found that CcpA regulates central carbon metabolism, including the EMP pathway, gluconeogenesis, and the PPP in *S. bovis* S1. Additionally, glucose and CcpA likely influence pyruvate fermentation, directing it toward lactate or formate production by modulating FBP concentrations. These findings underscore the regulatory roles of glucose concentration and CcpA in metabolic pathways, particularly in fermentation and energy metabolism in *S. bovis* S1.

## INTRODUCTION

High-concentrate diets increase the risk of suffering from rumen organic acid accumulation, leading to barrier destruction, inflammation, and oxidation (1, 2). Ruminants may suffer from rumen acidosis when fed high-concentrate diets due to the higher proliferation and overproduction of lactate by *Streptococcus bovis *(3–5).

Catabolite control protein A (CcpA) regulates the transcription of lactate dehydrogenase (LDH) and pyruvate formate-lyase (PFL) in *S. bovis*, depending on glucose concentration (6), which could regulate lactic acid generation. Previous studies showed the role of lactic acid over volatile fatty acids due to their lower pKa value in the development of subacute ruminal acidosis (SARA) or ruminal acidosis (7, 8). Therefore, regulating lactic acid production or its metabolites could potentially mitigate the risk of ruminal acidosis. Ruminal bacteria play a pivotal role in carbon-hydrogen metabolism and utilization (9). Metagenomic studies have provided insights into the carbohydrate-active enzyme (CAZyme) profile of ruminal bacteria (10). *S. bovis*, as a key rumen bacterium with excellent tolerance to low-pH environments, responds to high-concentrate diets with rapid reproduction and increased lactic acid production in the final fermentation fluid (3–5). Whole-genome sequencing results have elucidated the encoding ability of *S. bovis* for CAZymes (11). Carbon-hydrogen compounds would degrade to glucose, which is fermented to pyruvate and subsequently utilized by LDH or PFL in *S. bovis* (4). The efficient conversion of pyruvate to lactic acid is a more significant pathway than its conversion to formic acid, potentially leading to lactic acid accumulation (12, 13). Therefore, leading the fermentation model disproportionately or utilizing lactic acid could reduce the potential risk of lactic acid accumulation and increase carbohydrate utilization. Intervening in the carbon metabolism process in *S. bovis* could serve as a potential regulatory point to reduce lactic acid accumulation in high-concentrate diets (14).

CcpA, acting as a transcription factor, plays a pivotal role in regulating the expression of catabolic and metabolic genes of carbohydrates in bacteria (14–18). Typically, CcpA binds to target genes via catabolite response element sequences located upstream or within promoters, thereby activating or inhibiting transcription (19–22). Previous studies have elucidated CcpA’s role in regulating glucose metabolism processes, including the Embden-Meyerhof-Parnas (EMP) pathway, the tricarboxylic acid (TCA) cycle, and pyruvate fermentation (23–25). These processes are modulated by glucose concentration through regulation of gene expression levels mediated by CcpA, leading to the generation of secondary carbon sources such as fructose-1,6-bisphosphate (FBP) and dihydroxyacetone phosphate (DHAP). Additionally, these secondary carbon sources synergistically mediate glucose metabolism in conjunction with CcpA (12, 25). The type of substrate also mediates bacterial growth through CcpA expression (26). These regulatory mechanisms ensure bacterial adaptability to varying substrates. Mutations or knockouts of the CcpA gene have been shown to alter the fate of pyruvate and decrease lactic acid production (27). Furthermore, the expression levels of LDH and PFL are also regulated following *ccpA* mutation or knockout (6). Therefore, CcpA emerges as a potential regulatory point in *S. bovis* to reduce lactic acid production.

Our previous findings revealed the limited growth velocity of *S. bovis* S1 and distinct fermentation outcomes following *ccpA* knockout (6, 27). Particularly, under high-glucose concentrations, lactic acid levels decreased while concentrations of formic acid and acetic acid increased (6, 27). Glucose and CcpA mediated the EMP pathway and pyruvate fermentation. Transcriptome analysis corroborated these findings, suggesting differential gene expression levels related to carbon utilization and altered metabolic models (6). The current study aims to investigate energy metabolites in *S. bovis* S1 to elucidate metabolic shifts under conditions of glucose limitation or excess, with or without *ccpA* knockout.

## RESULTS

### Organic acid profiles between excess or limited glucose and with or without *ccpA* knockout in *S. bovis* S1

**Fig 1:**
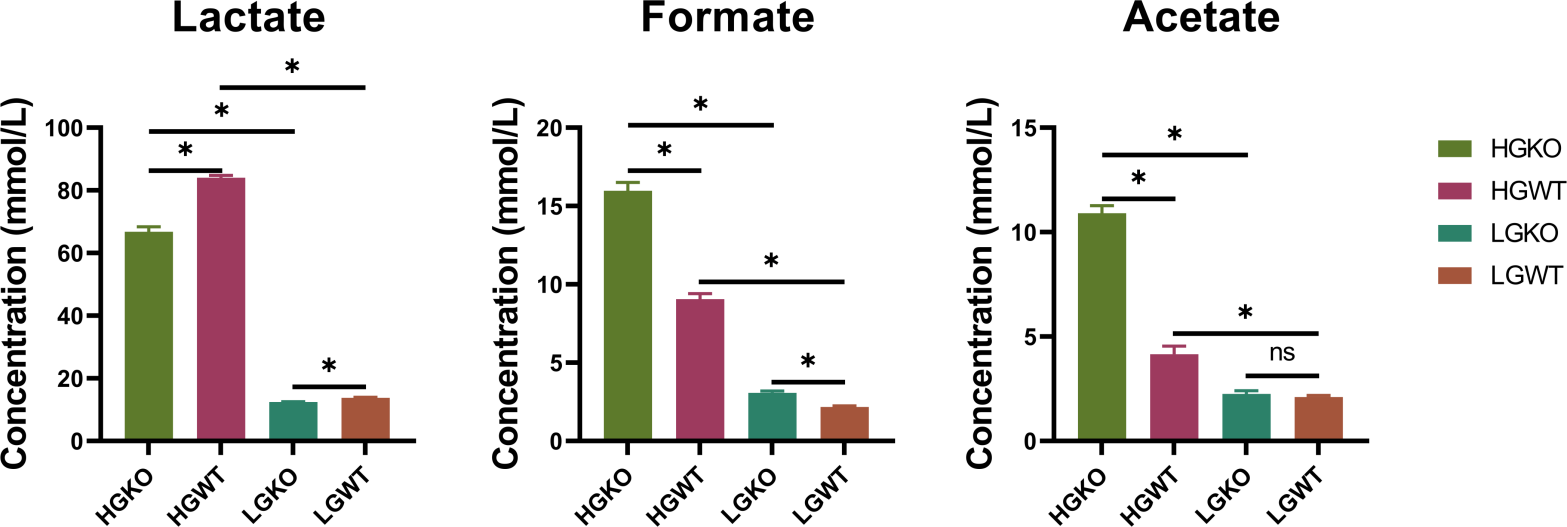
Organic acid compositions among HGWT, HGKO, LGWT, and LGKO groups. CcpA, catabolite control protein A; HGKO, *ccpA* knockout *S. bovis* S1 incubation with high-glucose concentration; HGWT, *S. bovis* S1 wild-type incubation with high-glucose concentration; LGKO, *ccp*A knockout *S. bovis* S1 incubation with low-glucose concentration; LGWT, *S. bovis* S1 wild-type incubation with low-glucose concentration; ns, not significant. *P < 0.05.

Organic acid profiles were previously reported by Jin et al. (6). Briefly, limited glucose reduced the concentrations of lactate, formate, and acetate compared to excess glucose treatment, regardless of *ccpA* knockout (*P* < 0.05, *S. bovis* S1 wild-type incubation with low-glucose concentration [LGWT] vs *S. bovis* S1 wild-type incubation with high-glucose concentration [HGWT], *ccpA* knockout *S. bovis* S1 incubation with low-glucose concentration [LGKO] vs CcpA knockout *S. bovis* S1 incubation with high-glucose concentration [HGKO]; Fig. 1). *ccpA* knockout decreased lactate concentration while increasing formate concentration under both excess and limited glucose conditions. Additionally, acetate concentration increased following *ccpA* knockout under excess glucose conditions but showed no significant difference under limited glucose conditions (*P* < 0.05, HGKO vs HGWT, LGKO vs LGWT; Fig. 1).

### Cellular energy metabolite profiles between excess or limited glucose and with or without *ccpA* knockout in *S. bovis* S1

**Fig 2:**
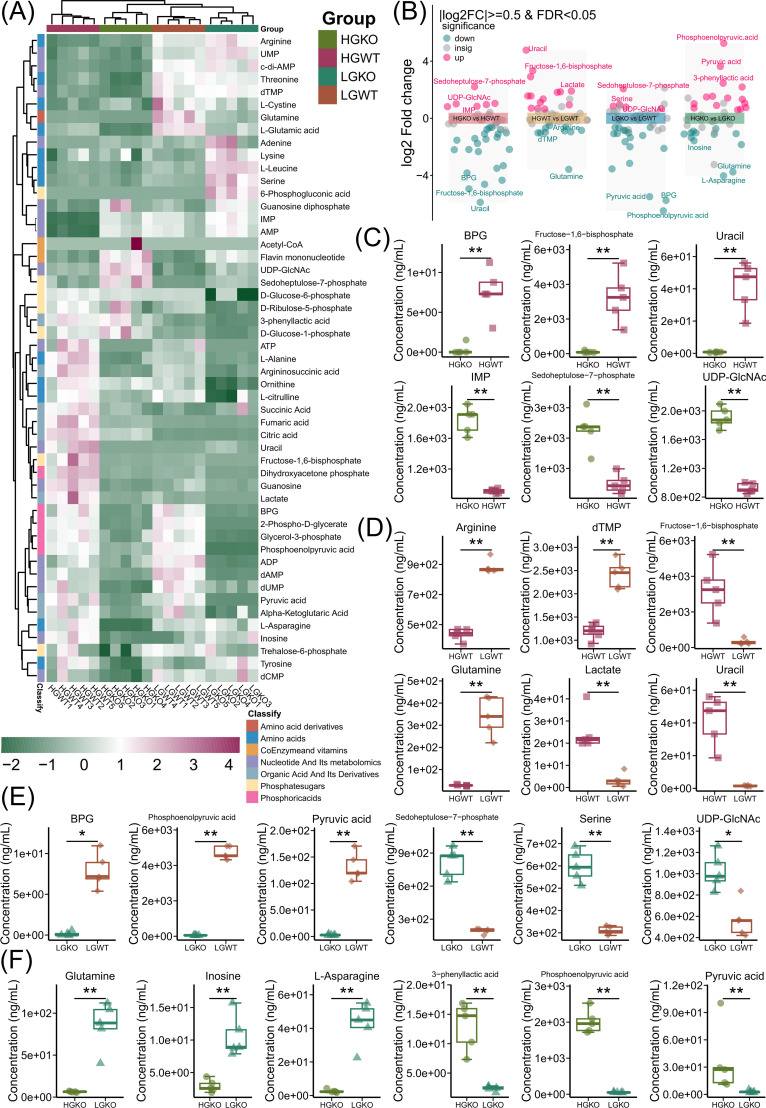
Cellular energy metabolic profiles among HGWT, HGKO, LGWT, and LGKO groups. (A) A heatmap showing the cellular metabolic profiles among HGWT, HGKO, LGWT, and LGKO groups. (B) Multi-volcano plot showing the enrichment and the top three metabolites based on |log2FC| of ≥0.5 and *P* value of <0.05 in the comparison between HGKO and LGKO (HGKO vs HGWT), the comparison between HGWT and LGWT (HGWT vs LGWT), the comparison between LGKO and LGWT (LGKO vs LGWT), and the comparison between HGKO and LGKO (HGKO vs LGKO). (C) Box plot showing the top three metabolites in HGKO vs HGWT. (D) Box plot showing the top three metabolites in HGWT vs LGWT. (E) Box plot showing the top three metabolites in LGKO vs LGWT. (F) Box plot showing the top three metabolites in HGKO vs LGKO. CcpA, catabolite control protein A; HGKO, *ccpA* knockout *S. bovis* S1 incubation with high-glucose concentration; HGWT, *S. bovis* S1 wild-type incubation with high-glucose concentration; LGKO, *ccpA* knockout *S. bovis* S1 incubation with low-glucose concentration; LGWT, *S. bovis* S1 wild-type incubation with low-glucose concentration. *P < 0.05, **P < 0.01.

Among the four treatments, a total of 51 energy metabolites were both qualitatively and quantitatively assessed. These metabolites were categorized into seven types: amino acid derivatives, amino acids, coenzymes and vitamins, nucleotides and their metabolites, organic acids and their derivatives, phosphate sugars, and phosphoric acids (Fig. 2A). Notably, HGWT (HGWT group) was enriched in ATP, L-alanine, argininosuccinic acid, ornithine, L-citrulline, succinic acid, fumaric acid, citric acid, uracil, fructose-1,6-bisphosphate, dihydroxyacetone phosphate, guanosine, lactate, and pyruvic acid, which exhibited higher abundance (Fig. 2A; Fig. S2 and Fig. S3A). Conversely, flavin-mononucleotide, UDP-GlcNAc, sedoheptulose-7-phosphate, D-ribulose-5-phosphate, 3-phenyllactic acid, and D-glucose-1-phosphate were more abundant in HGKO (HGKO group) (Fig. 2A; Fig. S2 and Fig. S3A). Threonine, dTMP, L-cystine, glutamine, L-glutamic acid, ADP, dAMP, and dUMP were found to be elevated in LGWT (LGWT group) (Fig. 2A; Fig. S2 and Fig. S3A), whereas arginine, UMP, c-di-AMP, adenine, lysine, L-leucine, serine, and 6-phosphogluconic acid exhibited higher levels in HGKO (HGKO group) (Fig. 2A; Fig. S2 and Fig. S3A).

### Enrichment energy metabolites compared between excess or limited glucose and with or without *ccpA* knockout in *S. bovis* S1

Glucose excess or limitation, along with *ccpA* knockout or its presence in *S. bovis* S1, revealed distinct cellular energy metabolite profiles (Fig. 2A). Using |log2FC| > 1 and *P* < 0.05 criteria, the enrichment of energy metabolites was determined by comparing different groups (Fig. 2B). The comparison between HGKO and HGWT groups revealed 6 enriched metabolites in HGKO and 23 enriched metabolites in HGWT. Among these, sedoheptulose-7-phosphate, UDP-GlcNAc, and inosine monophosphate (IMP) were among the top three cellular energy metabolites in HGKO, while 1,3-bisphosphoglycerate (BPG), fructose-1,6-bisphosphate, and uracil were the top three cellular energy metabolites in HGWT (Fig. 2B and C). When comparing HGWT and LGWT groups, 14 metabolites were enriched, with uracil, fructose-1,6-bisphosphate, and lactate being the top three cellular energy metabolites in HGWT, while 10 metabolites were enriched, with glutamine, dTMP, and arginine being the top three cellular energy metabolites in LGWT (Fig. 2B and D). In the comparison between LGKO and LGWT groups, 5 metabolites were upregulated, with sedoheptulose-7-phosphate, serine, and UDP-GlcNAc being the top three cellular energy metabolites, while 18 metabolites were downregulated, with phosphoenolpyruvic acid, BPG, and pyruvic acid being the top three cellular energy metabolites in LGKO (Fig. 2B and E). Finally, comparing HGKO and LGKO groups, 15 metabolites were upregulated, with the top three cellular energy metabolites being phosphoenolpyruvic acid, pyruvic acid, and 3-phenyllactic acid in HGKO, while 12 metabolites were upregulated, with L-asparagine, glutamine, and inosine being the top three cellular energy metabolites in LGKO (Fig. 2B and F).

### Multi-statistical analyses for energy metabolites between excess or limited glucose and with or without *ccpA* knockout in *S. bovis* S1

**Fig 3:**
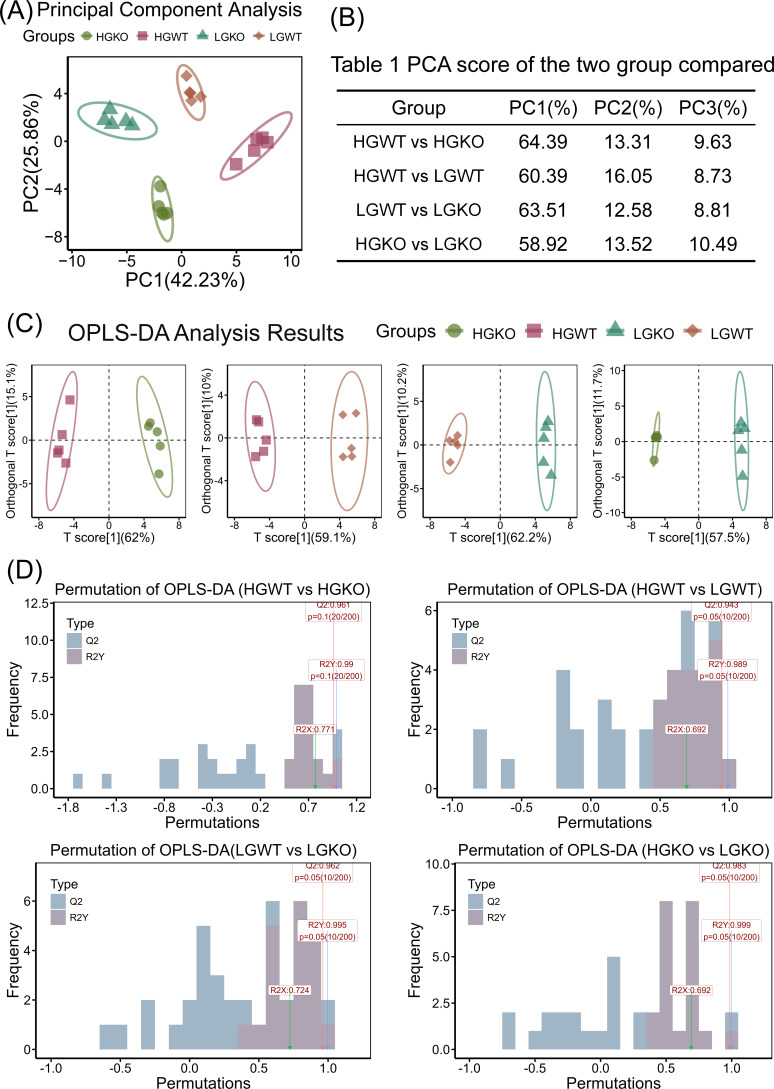
Multi-statistical analyses for energy metabolites between excess or limited glucose and with or without *ccpA* knockout in *S. bovis* S1. (A) PCA showing the differences among HGWT, HGKO, LGWT, and LGKO groups. (B) PC1, PC2, and PC3 scores of HGWT vs HGKO, HGWT vs LGWT, LGWT vs LGKO, and HGKO vs LGKO. (C) OPLS-DA showing the differences in HGWT vs HGKO, HGWT vs LGWT, LGWT vs LGKO, and HGKO vs LGKO. (D) Permutation of OPLS-DA for HGWT vs HGKO, HGWT vs LGWT, LGWT vs LGKO, and HGKO vs LGKO. CcpA, catabolite control protein A; HGKO, *ccpA* knockout *S. bovis* S1 incubation with high-glucose concentration; HGWT, *S. bovis* S1 wild-type incubation with high-glucose concentration; LGKO, *ccpA* knockout *S. bovis* S1 incubation with low-glucose concentration; LGWT, *S. bovis* S1 wild-type incubation with low-glucose concentration; PCA, principal component analysis.

Based on cellular energy metabolite profiles among the four treatments, principal component analysis (PCA) results revealed significant differences in metabolite composition. PC1 and PC2 contributed 42.23% and 25.86%, respectively, to the variation among samples (Fig. 3A). The analysis indicated that 28 metabolites exceeded the average contribution value of PC1, while 20 metabolites exceeded the average contribution value of PC2 (Fig. S1). The PC1, PC2, and PC3 values for HGWT vs HGKO, HGWT vs LGWT, LGWT vs LGKO, and HGKO vs LGKO are presented in Fig. 3B, with PC1 + PC2 values ranging from 72.44% to 77.70% across the four groups. The contribution values of PC1, PC2, and PC3 are shown in Fig. 3B. Furthermore, an OPLS-DA analysis, aimed at maximizing interval grouping differences, was conducted. In the comparison between HGWT and HGKO groups, OPLS-DA predicted and orthogonal principal components explained 62% and 15.1% of the variation, respectively (Fig. 3C). Similar analyses revealed that in HGWT vs LGWT, LGWT vs LGKO, and HGKO vs LGKO groups, predicted principal components explained 59.1%, 62.2%, and 57.5% of the variation, respectively, while orthogonal principal components explained 10%, 10.2%, and 11.7% of the variation, respectively (Fig. 3C). The four OPLS-DA analyses showed *Q*2 values exceeding 0.9, indicating robust predictive performance. Additionally, permutation plots displayed *P* values of 0.05 for both *Q*2 and R2Y at bin 10/200 (Fig. 3D).

### Differential metabolite screen and function prediction

**Fig 4:**
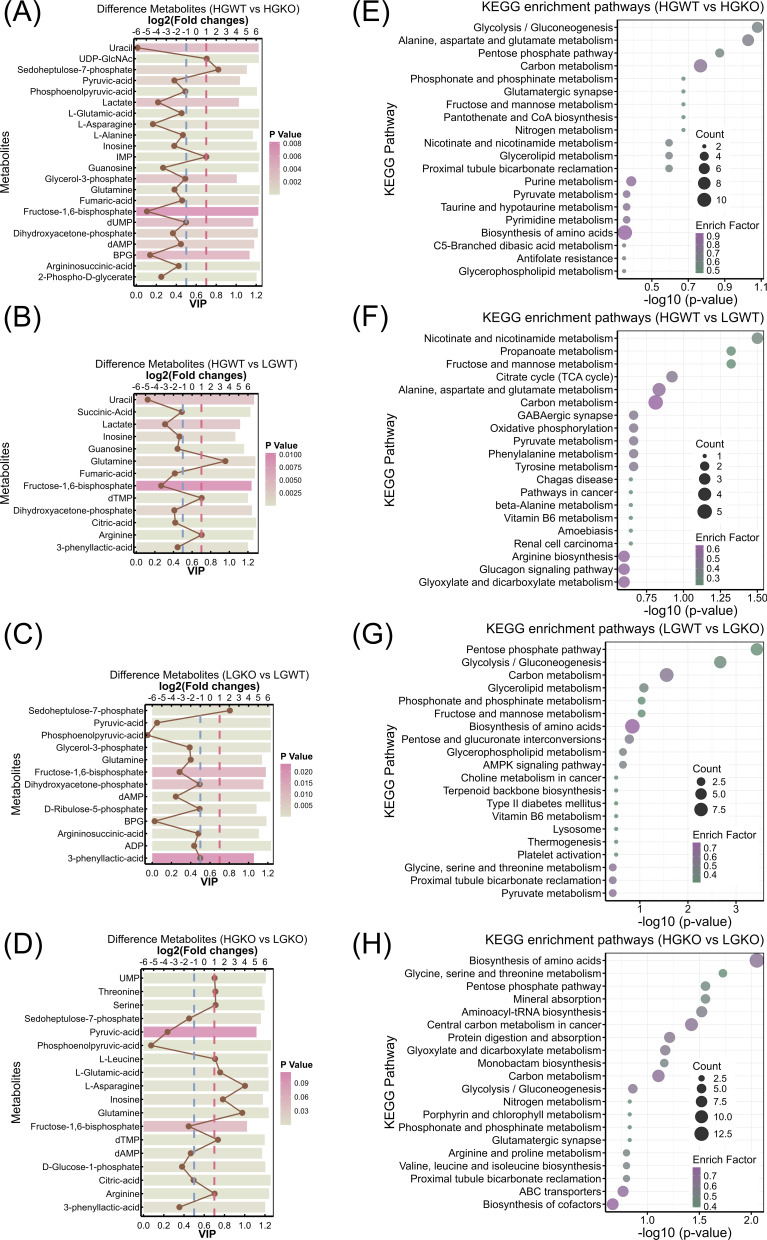
(A–D) Bar with line plot showing the differences in metabolites based on |log2FC| of >1 and VIP of >1 in HGWT vs HGKO, HGWT vs LGWT, LGWT vs LGKO, and HGKO vs LGKO. (E–H) Bubble plot showing the KEGG enrichment pathways based on the differences in metabolites in HGWT vs HGKO, HGWT vs LGWT, LGWT vs LGKO, and HGKO vs LGKO. CcpA, catabolite control protein A; HGKO, *ccpA* knockout *S. bovis* S1 incubation with high-glucose concentration; iHGWT, *S. bovis* S1 wild-type incubation with high-glucose concentration; KEGG, Kyoto Encyclopedia of Genes and Genomes; LGKO, *ccpA* knockout *S. bovis* S1 incubation with low-glucose concentration; LGWT, *S. bovis* S1 wild-type incubation with low-glucose concentration.

Based on variable importance in projection (VIP) > 1 and |log2FC| > 1 criteria, differential metabolites were screened between selected treatments. In the comparison between HGWT and HGKO groups, 23 metabolites showed differences, with 3 upregulated and 20 downregulated (Fig. 4A). These 23 metabolites were enriched in pathways such as glycolysis/gluconeogenesis, alanine, aspartic acid and glutamate metabolism, pentose phosphate pathway (PPP), carbon metabolism, purine metabolism, and biosynthesis of amino acids (Fig. 4E). Comparing HGWT with LGWT, 3 metabolites were upregulated and 10 were downregulated (Fig. 4B). These 13 differential metabolites were involved in pathways including niacin and nicotinamide metabolism, propionate metabolism, fructose and mannose metabolism, TCA cycle, alanine, aspartate and glutamate metabolism, carbon metabolism, arginine biosynthesis, glucagon signaling pathway, glyoxylate, and dicarboxylate metabolism (Fig. 4F). Between LGWT and LGKO groups, 17 differential metabolites were identified, with 13 visualized due to their significant changes. Four metabolites with fold change at 0 or infinity were excluded from visualization, while 2 metabolites (6-phosphogluconic acid not shown) were upregulated and 15 were downregulated (ATP, lactic acid, and 2-phosphate-D-glyceroic acid not shown) (Fig. 4C). These metabolites were enriched in pathways such as the pentose phosphate pathway, glycolysis/gluconeogenesis pathway, carbon metabolism, and glyceride metabolism (Fig. 4G). Between the HGKO and LGKO groups, 21 differential metabolites were observed, with 11 upregulated in HGKO (6-phosphogluconic acid not shown) and 10 upregulated in LGKO (lactic acid and 6-phosphogluconic acid not shown) (Fig. 4D). These metabolites were involved in pathways such as biosynthesis of amino acids, pentose phosphate pathway, carbon metabolism, glycolysis/gluconeogenesis, ABC transporters, and biosynthesis of cofactor pathway (Fig. 4H).

### Biomarker metabolites identified between excess or limited glucose and with or without *ccpA* knockout in *S. bovis* S1

**Fig 5:**
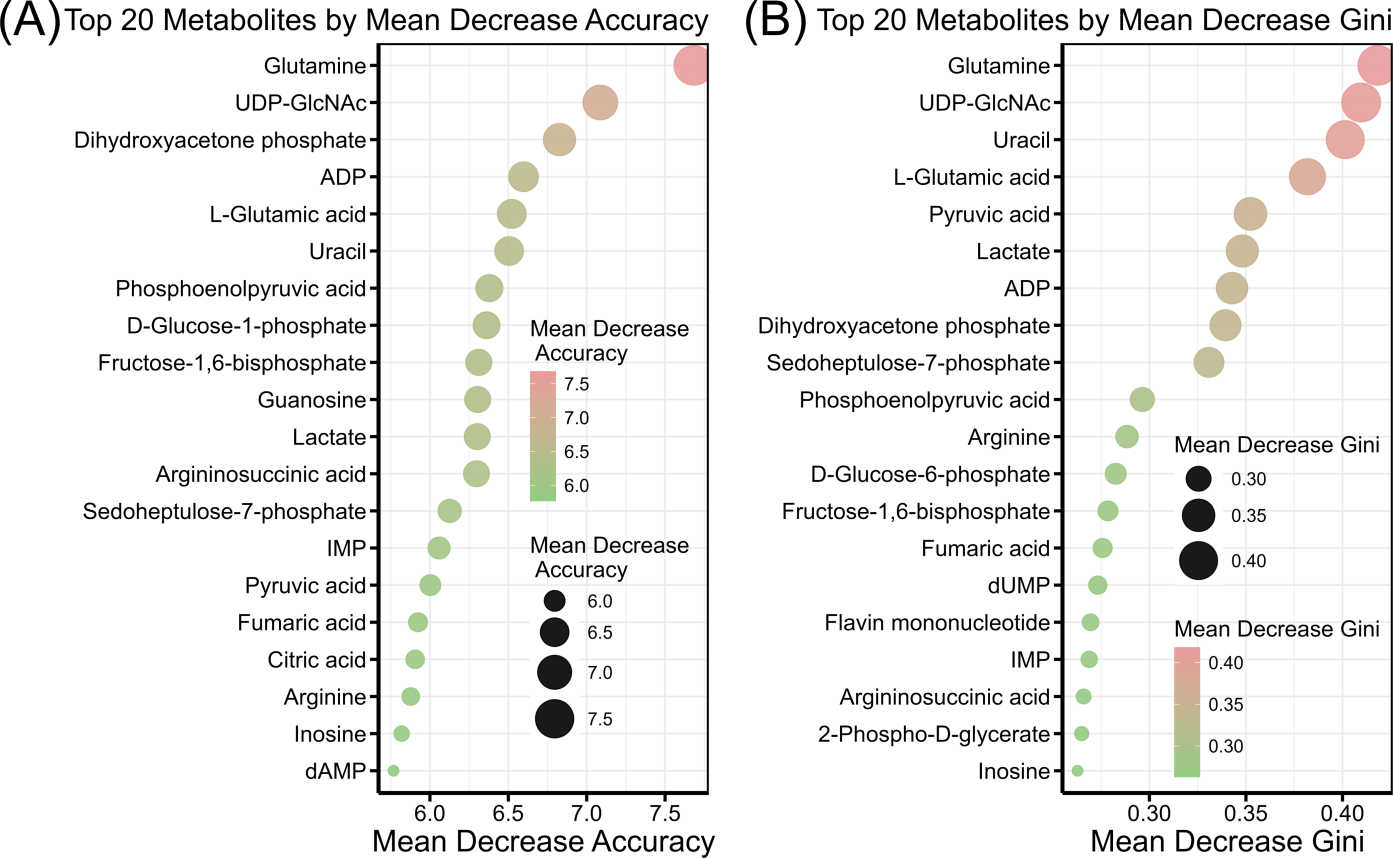
Biomarkers of cellular energy metabolites identified by the random forest algorithm. (**A**) Importance based on mean decrease accuracy. (**B**) Importance based on mean decrease in Gini.

Biomarker metabolites were identified between excess and limited glucose conditions, as well as in the presence or absence of *ccpA* knockout in *S. bovis* S1. These results demonstrate that glucose and CcpA modulate cellular energy metabolism with various changes. The random forest algorithm was used to identify biomarkers in *S. bovis* S1, and glutamine and UDP-GlcNAc were found to have a mean decrease accuracy greater than 7.0 and a mean decrease Gini greater than 0.40 (Fig. 5A and B). Glutamine and UDP-GlcNAc showed different levels and significant differences across the four experimental groups (*P* < 0.05, Fig. S2). Therefore, glutamine and UDP-GlcNAc can serve as cellular energy metabolite biomarkers under the dual regulation of glucose and CcpA in *S. bovis* S1.

## DISCUSSION

A high-concentrate diet leads to lactate accumulation and *S. bovis* bloom in the rumen, potentially increasing the risk of SARA occurrence (5, 7, 8). CcpA has been reported to regulate carbon metabolism direction (26). Based on lactate, formate, and acetate concentrations, limited glucose availability resulted in lower fermentation activity, which was further reduced by *ccpA* knockout. Our previous research demonstrated that *ccpA* knockout in *S. bovis* induced differential gene expression related to carbon metabolism at the transcriptome level (6). In this study, we further evaluated differences in cellular energy metabolism profiles. Using liquid chromatography tandem mass spectrometry (LC-MS/MS) detection, we identified and quantified 51 selected cellular energy metabolites under conditions of glucose excess or limitation and with or without *ccpA* knockout. Based on PCA and OPLS-DA analysis, *S. bovis* S1 exhibited distinct cellular metabolite profiles; these profiles unraveled the crosstalk between glucose and CcpA. The differential and enriched metabolites elucidated the strategies employed by *S. bovis* S1 under conditions of glucose excess or limitation and with or without *ccpA* knockout, primarily focusing on carbon metabolism, amino acid metabolism, and nucleotide metabolomics.

In terms of carbon metabolism, Kyoto Encyclopedia of Genes and Genomes (KEGG) function prediction indicated involvement in the EMP pathway, the TCA cycle, and pyruvate metabolism. In this study, low-glucose levels induced lower lactate, formate, and acetate levels in fermentation fluid and reduced cellular concentrations of FBP and DHAP, consequently leading to diminished EMP flux. FBP was also found to decrease LDH expression levels while increasing PFL expression levels (28), and the substrate glucose concentration could reshape PFL and LDH expression levels (4). Additionally, DHAP inhibited PFL activity (12). However, cellular PEP and pyruvate concentrations were similar under different glucose concentrations (Fig. S3A). These changes interplay to influence the survival strategies and final pyruvate fermentation outcomes. Succinic acid, fumaric acid, and citric acid concentrations decreased under low-glucose conditions, indicating potential limitations in the flux of the citric cycle. The reduced carbon flux under low-glucose conditions consequently led to limited growth velocity (6). This might reflect a bacterial strategy under limited or fluctuating nutrient conditions, with sharply pulsed gene expression designed to prolong exponential growth while ultimately decreasing overall growth (29, 30). *ccpA* knockout resulted in decreased concentrations of 6-phosphogluconic acid, fructose-1,6-bisphosphate, D-glucose-6-phosphate, dihydroxyacetone phosphate, glycerol-3-phosphate, BPG, and phosphoenolpyruvic acid, leading to a lower sugar phosphate pool (Fig. S2 and Fig. S4A). This suggests that the *ccpA* knockout disrupted the EMP pathway. The fitted curve between cellular sugar phosphate levels and lactate production (Fig. S4B) highlights the importance of the EMP pathway in lactate generation within the cell. *ccpA* knockout might decrease the sugar phosphate in EMP flux to reduce lactic acid production. Pyruvate kinase and phosphofructokinase are two key enzymes in the EMP pathway, and their expression levels were decreased according to transcriptome results (6). *ccpA* knockout may regulate the activity of these enzymes to modulate EMP flux. Similar observations of EMP flux alterations have been reported after *ccpA* knockout in *Staphylococcus aureus* (31). The fate of pyruvate includes metabolism to lactic acid, formic acid, acetic acid, and acetyl-CoA. In this study, we observed differences in the composition of organic acids in the fermentation fluid following *ccpA* knockout. However, acetyl-CoA levels were almost undetectable in *S. bovis* S1, likely due to anaerobic incubation conditions, and the presence of succinic acid, fumaric acid, and citric acid indicates that fermentation is the primary metabolic pathway for pyruvate under anaerobic conditions, with lactate fermentation being a more efficient means to generate ATP compared to formic acid and acetic acid (12, 13). *ccpA* coordinated oxygen and carbon metabolism in *Lactococcus lactis* (32). The absence of oxygen may cause carbon metabolism to primarily focus on the EMP pathway and pyruvate fermentation in *S. bovis* S1. Following *ccpA* knockout, a classic observation is a decrease in lactic acid production in fermentation fluid (27); indeed, the decrease in lactic acid in the fermentation fluid and cellular metabolites was also observed in this study. LDH expression levels were found to decrease in our previous study (6). *ccpA* knockout results in reduced flux through the EMP pathway and influences the direction of pyruvate fermentation. In short, glucose and CcpA regulate pyruvate production through the EMP pathway. Glucose concentration determines the carbon flux into the EMP, while CcpA governs overall EMP flux. CcpA also influences the expression levels of *ldh* and *pfl* (6). However, current research has not confirmed whether CcpA directly targets *ldh* or *pfl*. Instead, an indirect mechanism may mediate *ldh* and *pfl* expression. FBP has been correlated with *ldh* and *pfl* expression levels (28), suggesting that glucose and CcpA may regulate FBP concentration as a means to modulate LDH and PFL activity, ultimately affecting lactate and formate production. Further research should focus on the enzymatic or gene regulatory mechanisms controlled by CcpA.

In terms of amino acid metabolism, glutamine was identified as the biomarker in the energy metabolism network for *S. bovis* S1 to adapt to the glucose and CcpA environment. Low-glucose levels induce higher cellular concentrations of glutamine and L-glutamic acid. Glutamine is involved in both the EMP pathway and gluconeogenesis, as well as in crosstalk between carbon and nitrogen metabolism (33). Glutamine and L-glutamic acid, being common amino acids, can be obtained by degrading protein in medium. They play a crucial role in microbial protein and amino acid synthesis (34). The lower cellular concentration of alpha-ketoglutaric acid, as an intermediate product, suggests that the amino acid synthesis via glutamine might be downregulated. In a low-glucose medium, the priority of glutamine is to generate glucose to alleviate glucose deficiency. CcpA has been shown to inhibit amino acids as secondary carbon sources (35). *ccpA* knockout reduces the concentration of alpha-ketoglutaric acid, glutamine, and L-glutamic acid within bacteria. Additionally, *ccpA* knockout also regulated the expression level of glutamine synthetase (GS). Transcriptome results indicate decreased expression levels of *gltAB*, which are the operons of GS, following *ccpA* knockout (6). Thus, CcpA disruption interferes with glutamine synthesis and the utilization of glutamine to generate glucose under low-glucose conditions. Glutamic acid plays a central role in amino acid amino transfer, facilitating the transfer of other amino acids to mitigate deficiencies. In this study, the concentrations of threonine, cystine, ornithine, and alanine decreased after *ccpA* knockout. A similar downregulation of alanine concentration was observed in *Bacillus globulus* lacking CcpA (36). CcpA might mediate the synthesis of these amino acids via L-glutamic acid. However, the profiles of serine, lysine, tyrosine, arginine, leucine, citrulline, and asparagine showed variations in *ccpA* knockout under different glucose concentrations. *ccpA* mutations have also been noted to lead to decreased leucine, lysine, tyrosine, and asparagine in *Lysinibacillus sphaericus* (36). Glucose depletion rates may influence CcpA function, leading to these variations. In this study, sampling was conducted at an OD_600_ value of 0.6 for HGWT and HGKO and at an OD_600_ value of 0.2 for LGWT and LGKO. *ccpA* knockout resulted in similar glucose consumption rates under low-glucose conditions but slowed glucose consumption under high-glucose concentrations. Both glucose and CcpA have been shown to interact in *Clostridium difficile* (37). The presence of glucose may interact with *ccpA* knockout results, thus influencing the metabolism of these amino acids in *S. bovis* S1.

In nucleotide metabolism, UDP-GlcNAc is another identified biomarker; it serves as an essential precursor for the cell wall (38). *ccpA* knockout increases cellular UDP-GlcNAc levels, regardless of whether the environment has high- or low-glucose concentrations. Under low-glucose conditions, the cellular UDP-GlcNAc level decreases, irrespective of the presence or absence of CcpA. Preliminary growth data show a detrimental effect due to the loss of CcpA; however, this effect is not influenced by the glucose concentration in the environment (6). This suggests a steady survival strategy for *S. bovis* S1 following *ccpA* knockout. Purines and pyrimidines play a vital role in cell survival (39). In this study, these two types of nucleotides are differentially affected by the medium’s glucose concentration. Decreased D-ribulose-5-phosphate and sedoheptulose-7-phosphate levels indicate limited PPP activity. The presence of sufficient glutamine and asparagine suggests growth restriction of *S. bovis* S1, potentially disrupting purine nucleotide and pyrimidine nucleotide synthesis. Briefly, adenine and pyrimidine synthesis were strengthened, while guanosine synthesis was impaired. This inference is supported by cellular concentrations of IMP, AMP, adenine, UMP, inosine, and guanosine. However, cellular dAMP, dCMP, dTMP, and dUMP levels were higher in low-glucose conditions, indicating contradictory trends between deoxyribonucleotides and ribonucleotides. Our previous data indicate that during the platform period of low-glucose concentration, the OD_600_ value is lower than in high-concentration conditions (6). These deoxyribonucleotides and ribonucleotides were detected as free. The restricted growth indicates that increased deoxyribonucleotide levels in low glucose might be due to *S. bovis* S1 obtaining them from the medium not for hereditary substance synthesis but potentially compensating for ribonucleotide deficiencies. The ribonucleotides may be relatively regulated by *S. bovis* S1 to adapt to the low-glucose environment. We have previously verified limited growth velocity following *ccpA* knockout, particularly under high-glucose concentrations (6), which may lead to an imbalance in nucleotide metabolism. *ccpA* knockout decreases cellular D-ribulose-5-phosphate and increases sedoheptulose-7-phosphate concentration. Notably, 6-phosphogluconic acid was observed only in low-glucose conditions after *ccpA* knockout, while glutamine and asparagine were absent in high-glucose conditions after *ccpA* knockout. *ccpA* knockout disrupts the normal PPP, affecting cellular purine and pyrimidine synthesis differently under varying glucose concentrations. While the phenomenon of CcpA regulation of bacterial purine and pyrimidine metabolism has been observed in other bacteria at transcriptional and protein levels (40–42), research on metabolic levels remains scarce.

### Conclusion

**Fig 6:**
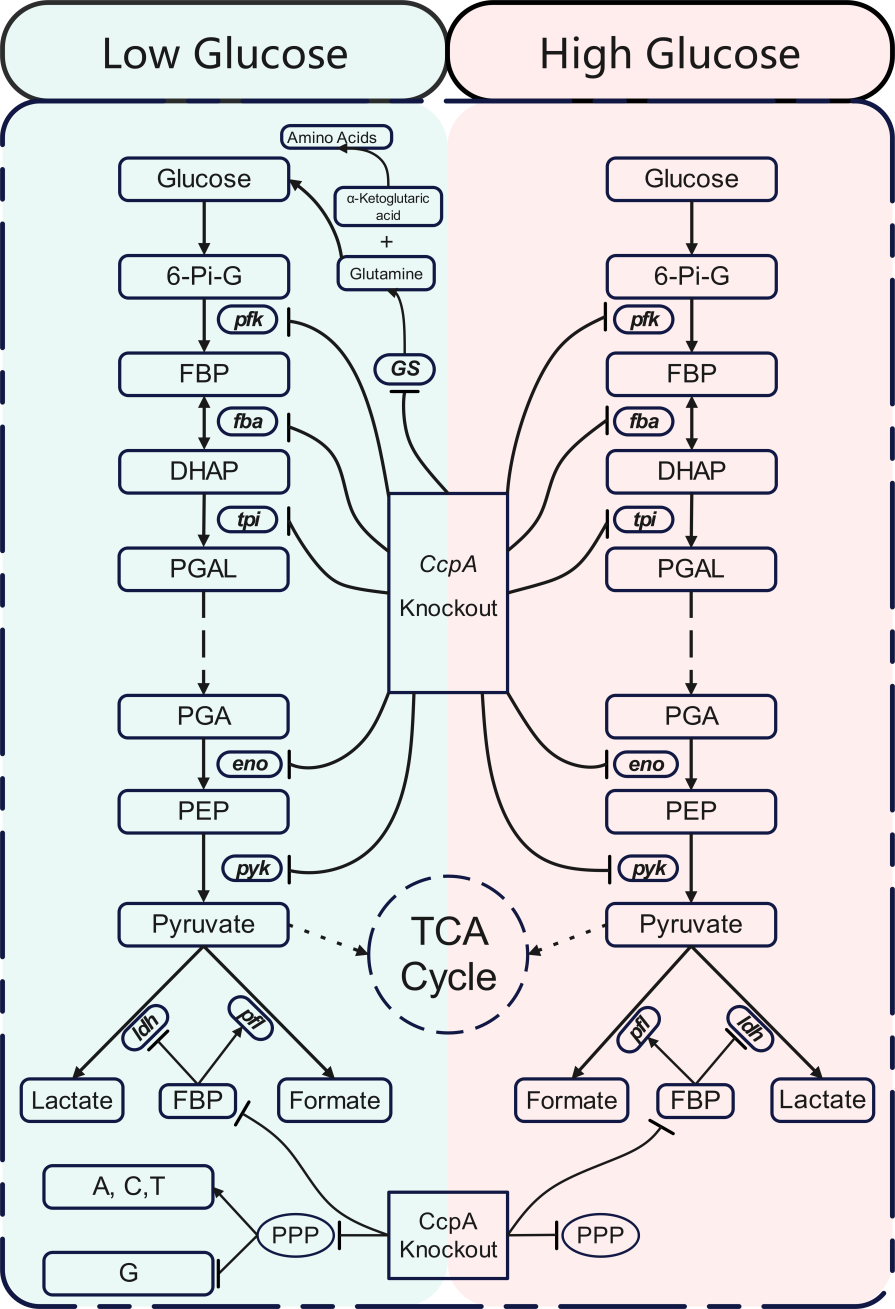
Schematic diagrams of glucose and *ccpA* interacting at cellular carbon metabolic flow in *S. bovis* S1. Low glucose limited or downregulated EMP, pyruvate fermentation, PPP, and G synthesis pathway but upregulated gluconeogenesis and the A, C, and T synthesis pathway compared to high glucose. *ccpA* knockout inhabited EMP, pyruvate fermentation to lactate by the FBP and PPP, and improved pyruvate fermentation to formate by FBP. TCA might not have occurred. CcpA, catabolite control protein A. Key metabolics: 6-Pi-G, 6-phosphoglucose; A, adenine; C, cytosine; DHAP, dihydroxyacetone phosphate; FBP, fructose-1,6-biphosphate; G, guanine; PEP, phosphoenolpyruvate; PGA, 3-phosphoglycerate; PGAL, glyceraldehyde-3-phosphate; T, thymine. Key enzymes: eno,enolase, converts 2-phosphoglycerate to PEP; fba, fructose-bisphosphate aldolase, splits FDP into DHAP and PGAL; GS, glutamine synthetase, catalyzes glutamine synthesis;idh, lactate dehydrogenase, converts pyruvate to lactate; pfl, pyruvate formate-lyase, converts pyruvate to formate, pfk, phosphofructokinase, catalyzes the conversion of 6-Pi-G to FDP; pyk, pyruvate kinase, converts PEP to pyruvate; tpi, triose-phosphate isomerase, interconverts DHAP and PGAL. Key metabolism pathway: PPP, pentose phosphate pathway; TCA, tricarboxylic acid. Metabolism flow: solid arrows indicate metabolic flow; solid arrows with bar indicate metabolic flow; long-dashed arrows represent ellipsis in metabolic flow; short-dashed arrows indicate metabolic flow that does not occur. Metabolism environment: light blue background denotes the low-glucose section, indicating a low-glucose environment with limited or downregulated EMP, pyruvate fermentation, PPP, and G synthesis metabolic activity, upregulated gluconeogenesis, A, C, and T synthesis compared to the high-glucose environment; light pink background denotes the high-glucose section, representing a high-glucose environment with elevated or normal metabolic activity.

As shown in Fig. 6, the knockout of the *ccpA* in *S. bovis* S1 under conditions of high or low-glucose concentration results in distinct cellular energy metabolite profiles. Further analysis reveals that low glucose restricts carbon flux, while *ccpA* knockout reduces flux through the EMP pathway and alters the direction of pyruvate fermentation, leading to decreased lactate production and significantly increased formate production. In low-glucose environments, glutamine serves to alleviate glucose deficiency. The interaction between glucose and CcpA may mediate the fate of amino acids differently. Low-glucose limits guanine synthesis but not that of adenine, cytosine, or thymine synthesis, while *ccpA* knockout disrupts both synthesis pathways through the PPP.

## MATERIALS AND METHODS

### Bacterial strains, media, and growth condition

*S. bovis* S1 used in this study was previously isolated from Sannen dairy goat in our laboratory by Chen et al. (5). The *ccpA* knockout of *S. bovis* S1 was described in Jin et al.’s study (27). The revived process and incubation process followed the method of Chen et al. (5). Briefly, the *S. bovis* S1 was revived by incubation with modified de Man, Rogosa, and Sharpe media in an anaerobic workstation (DG250; Don Whitley Scientific, England). The cultures (at exponential phase) were then incubated by mixing with a basal medium at 1:99. Then the 100 mL mixtures were added into 200 mL anaerobic serum bottles and sealed. These bottles were transferred to a thermostat shaker (TS-1102C; Bosheng Scientific Instrument Co., Yangzhou, China) under the conditions at 37°C and 2.86 × *g*. Ten percent NaOH was titrated to maintain the pH value of the mixtures at 6.5. The basal medium contained 0.45 g/L KH_2_PO_4_, 0.9 g/L NaCl, 0.9 g/L (NH_4_)_2_SO_4_, 0.12 g/L CaCl_2_⋅2H_2_O, 0.19 g/L MgSO_4_⋅7H_2_O, 1.0 g/L tryptone, 1.0 g/L yeast extract, and 0.6 g/L cysteine hydrochloride. Glucose solutions were added to the sterile basal medium at a final concentration of 5 or 50 mM, respectively.

### Experiment design

Twenty anaerobic serum bottles were randomly divided into four treatments and labeled with HGWT, HGKO, LGWT, and LGKO, with each treatment containing five bottles. HGWT was the wild-type *S. bovis* S1 incubated with 50 mM glucose; HGKO was *ccpA* knockout *S. bovis* S1 incubated with 50 mM glucose; LGWT was the wild-type *S. bovis* S1 incubated with 5 mM glucose; LGKO was *ccpA* knockout *S. bovis* S1 incubated with 5 mM glucose.

### Sample collection

The incubation fluid of each treatment was collected at the exponential growth phase, as previously described (6). Briefly, the OD_600_ of the HGWT and HGKO group reached 0.6 before being collected. The OD_600_ of the LGWT and LGKO groups reached 0.2 before being collected. The incubation fluid was centrifuged at 12,000 × *g* for 10 min at 4℃, and the supernatants were filtered using a 0.22 µm filter membrane for organic acids measurement. The sediment was washed with 10 mL cooled and sterile phosphate-buffered saline three times. After the last wash, the sediment was transferred to a 1.5 mL sterile Eppendorf tube, frozen in liquid nitrogen, and stored at −80℃.

### Fermentation fluid organic acids analysis

Organic acids (lactate, formate, and acetate) were measured using a high-performance liquid chromatographer system (Shimadzu, Japan) equipped with an Acclaim OA column (Sepax Carbomix H-NP) and a UV detector. The column temperature was maintained at 55°C; the mobile phase was 2.5 mM H_2_SO_4_, with a flow rate at 0.5 mL/min, and the UV detector was set at 210 nm.

### Sample processed for metabolic analysis

In this study, the metabolic process was conducted using the following method. Initially, samples were handled on ice, and the thawed sediment was treated with 500 µL of pre-cooled 80% methanol water solution. Subsequently, the mixture was rapidly frozen in liquid nitrogen for 5 min, thawed on ice, and vortexed for 2 min. This cycle was repeated three times to ensure comprehensive extraction. The extraction solution was then centrifuged at 10,000 × *g* for 10 min at 4°C, and 300 µL of supernatant was transferred into a new tube and stored for 30 min at −20°C. Subsequently, the solution was centrifuged again at 12,000 × *g* for 10 min at 4°C, and 200 µL of supernatant was collected for metabolic analysis.

### Chromatographic and mass spectrometry conditions

Fifty-seven metabolites involved in central carbon metabolites were selected for analysis. Metabolite analysis was conducted using ultra-high performance LC-MS/MS. An ExionLC AD system (Sciex, China), coupled with an ACQUITY UPLC BEH Amide column (1.7 µm, 100 mm × 2.1 mm), was employed to separate sample components. The mobile phase comprised two components: mobile phase A, consisting of 10 mM ammonium acetate and 0.3% ammonia aqueous solution, and mobile phase B, consisting of 90% acetonitrile aqueous solution. The mobile phase gradient was programmed as follows: the A:B ratio at 0–1.2 min was 5:95 (vol/vol); at 8.0 min, it was 30:70 (vol/vol); at 9.0–11.0 min, it was 50:50 (vol/vol); and at 11.1–15.0 min, it returned to 5:95 (vol/vol). The total mobile phase flow rate was maintained at 0.40 mL/min, and the column temperature was set to 40°C. A sample injection volume of 2 µL was used for analysis.

The qualitative and quantitative analyses of metabolites were performed by mass spectrometry (QTRAP 6500+, https://sciex.com.cn/). For qualitative analysis, the temperature of the electrospray ion source was set to 550℃; the voltage in positive ion mode was 5,500 V; the voltage in negative ion mode was −4,500 V; and the curtain gas was set at 35 psi. In Q-Trap 6500+, each ion pair was scanned and detected based on optimized declustering potential and collision energy. Quantitative analysis of the 57 metabolites was based on the external standard method. The information regarding qualitative and quantitative analyses is shown in Table S1.

### Statistical analyses and visualization

R software (version 4.3.3) was used for data statistical analysis and visualization. The pheatmap package was employed for heatmap plotting (43); the FactoMineR package was used for PCA (44); and the ropls package was used for OPLS-DA multi-analyses (45). Enriched metabolites were identified based on the following criteria: |log2FC| > 0.5 and *P* < 0.05. Biomarkers among the four groups were identified using the randomForest package. Visualization of the multi-volcano graph and the top three enriched metabolites was accomplished using the ggplot2 package, while statistical analysis of the top three enrichment metabolites was carried out using the ggpubr package (46, 47), with parameters set to “geom_pwc(method = “wilcox_test,” label = “p.signif,” p.adjust.method = “fdr,” size = 0.3, hide.ns = T).” Results were considered statistically significant at *P* < 0.05. The different metabolites between each pair of groups were screened based on |log2FC| > 1 and VIP > 1. These different metabolites were mapped to KEGG pathways for functional annotation.

## SUPPLEMENTAL MATERIAL

Supplemental figures spectrum.02150-24-s0001.docx Figures S1 to S4.

Table S1 spectrum.02150-24-s0002.xlsx Qualitative and quantitative information of the metabolites.

Table S2 spectrum.02150-24-s0003.xlsx Principal component axis contribution results among different conditions.

ASM does not own the copyrights to Supplemental Material that may be linked to, or accessed through, an article. The authors have granted ASM a non-exclusive, world-wide license to publish the Supplemental Material files. Please contact the corresponding author directly for reuse.
