# Supplementary material for: CcpA-mediated regulation of cellular energy metabolism in the ruminal bacterium Streptococcus bovis
Source: Microbiol Spectr. 2025 Jun 25;13(8):e02150-24. doi: 10.1128/spectrum.02150-24 (PMC12323346; doi:10.1128/spectrum.02150-24)
Supplement: Supplemental figures — Figures S1 to S4. [file spectrum.02150-24-s0001.docx]

**
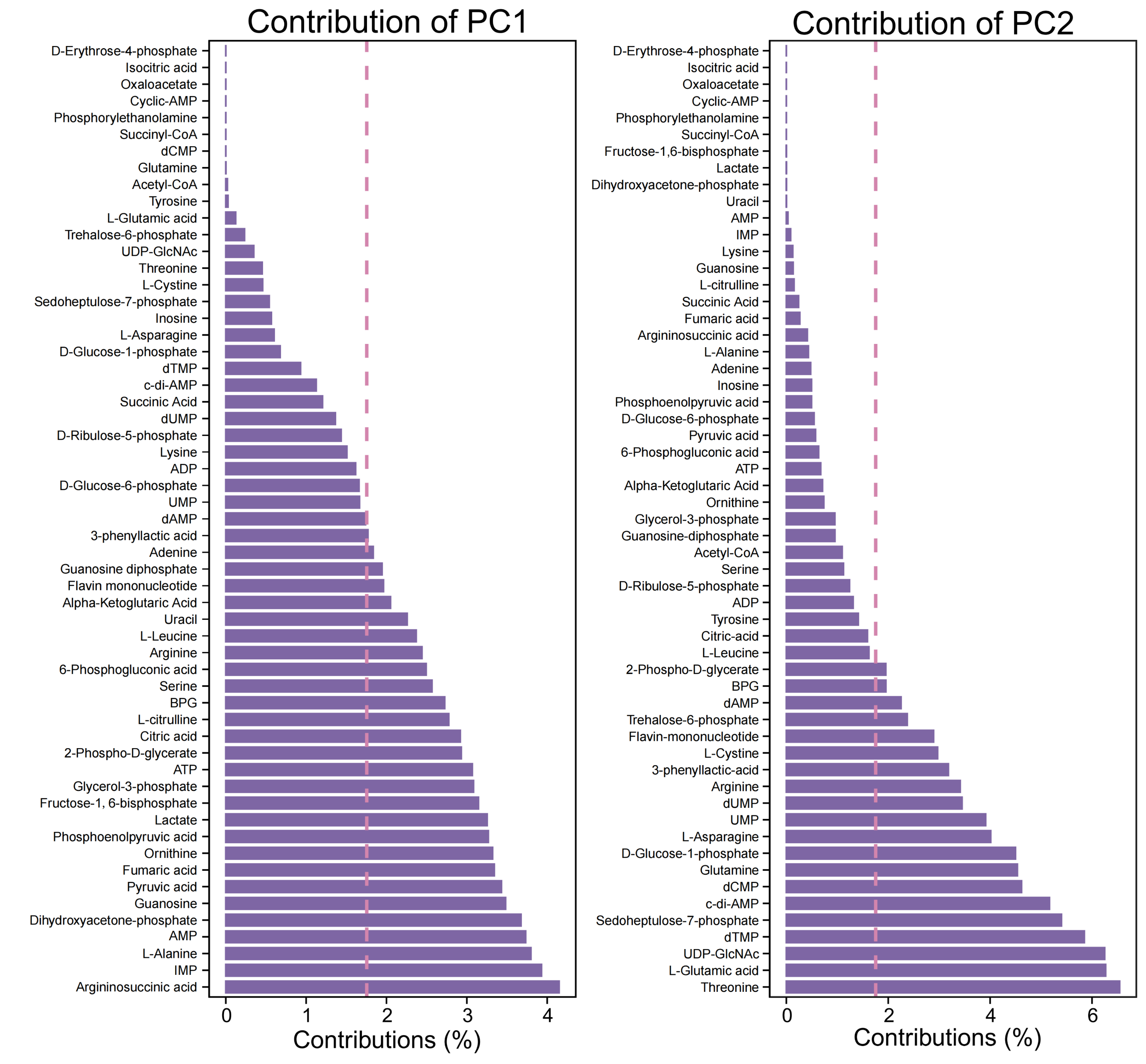
Fig. S1.** Contribution of metabolites for PC1 and PC2 under Principal Component Analysis (PCA) among HGWT, HGKO, LGWT, LGKO group. vertical line showing the average contribution in PC1 or PC2. HGWT: *Streptococcus bovis* S1 wild type incubation with high concentration glucose, HGKO: *CcpA* knockout *streptococcus bovis* S1 incubation with high glucose, LGWT: wild type *streptococcus bovis* S1 incubation with low glucose,LGKO: *CcpA* knockout *streptococcus bovis* S1 incubation with low glucose. *CcpA*: Catabolite control protein A.


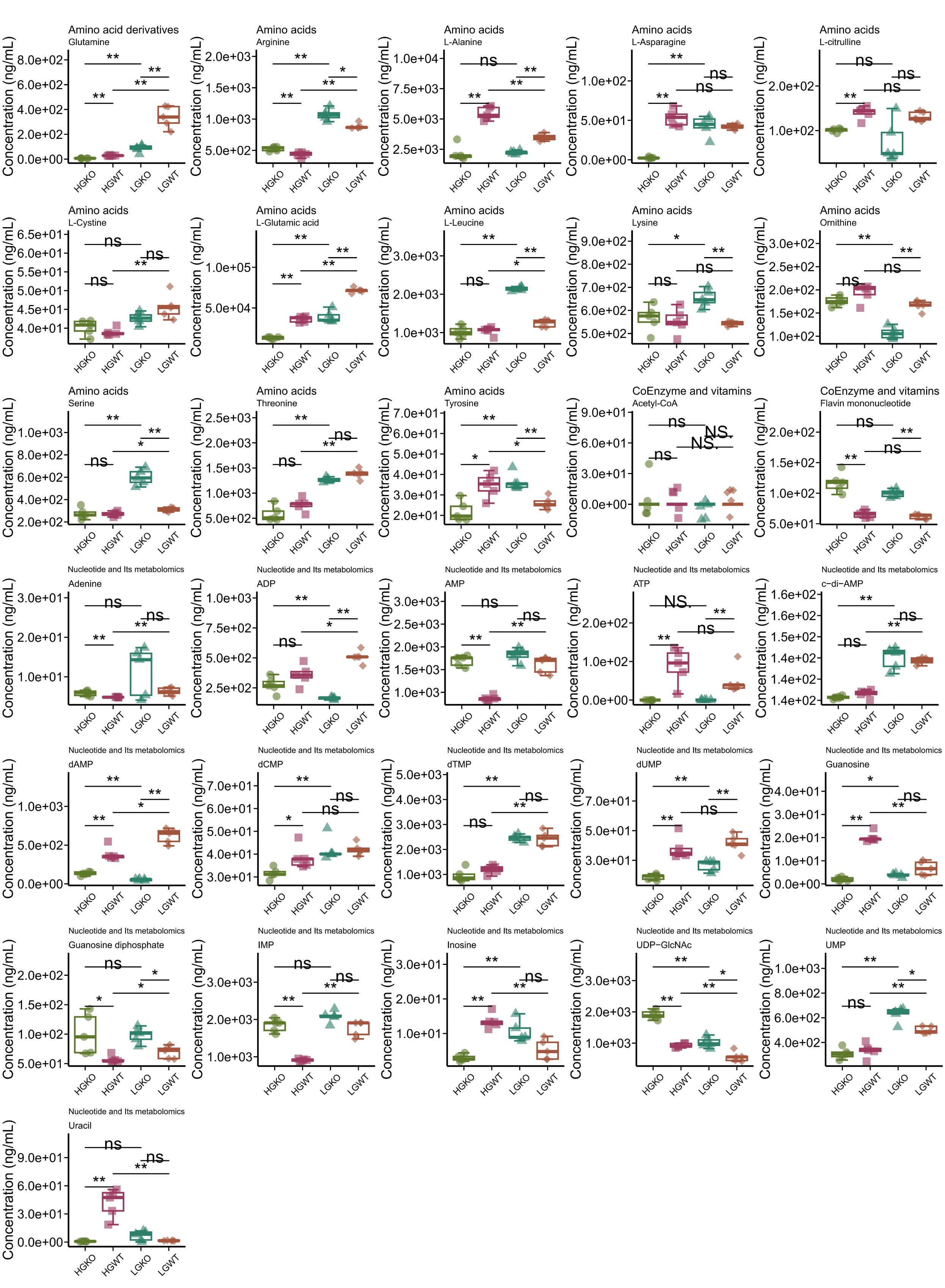


**Fig. S2.** Box plot showinng the metabolites differences which were classified into Amino acid derivatives, Amino acids, CoEnzymes and vitamins, Nucleotides and Its metabolomics among HGWT, HGKO, LGWT, LGKO group. Brackets including HGWT vs HGKO, HGWT vs LGWT, LGWT vs LGKO and HGKO vs LGKO. HGWT: *Streptococcus bovis* S1 wild type incubation with high concentration glucose, HGKO: *CcpA* knockout *streptococcus bovis* S1 incubation with high glucose, LGWT: wild type *streptococcus bovis* S1 incubation with low glucose, LGKO: *CcpA* knockout *streptococcus bovis* S1 incubation with low glucose. ** *p* < 0.01, * *p* < 0.05, ns *p*>0.05, NS. means can’t be compared the two group for didn’t detect the metabolite. *CcpA*: Catabolite control protein A.


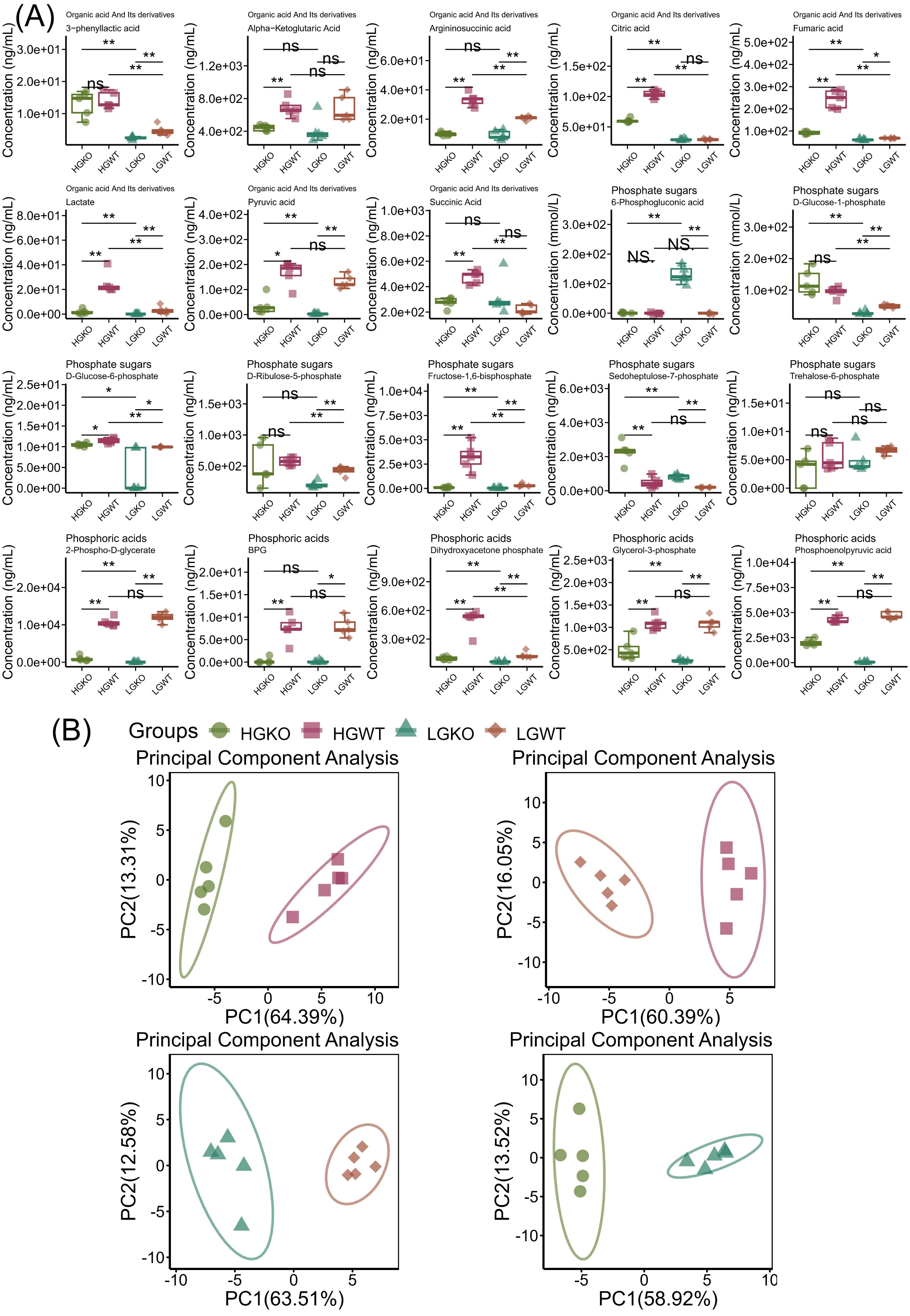


**Fig. S3(A).** Box plot showing the metabolites differences which were classified into Organic Acids and their Derivatives, Phosphate sugars, and Phosphoric acids among HGWT, HGKO, LGWT, LGKO group. Brackets including HGWT vs HGKO, HGWT vs LGWT, LGWT vs LGKO and HGKO vs LGKO. **Fig. S3(B).** Principal Component Analysis (PCA) showing the differences in HGWT vs HGKO, HGWT vs LGWT, LGWT vs LGKO and HGKO vs LGKO. HGWT: *Streptococcus bovis* S1 wild type incubation with high concentration glucose, HGKO: *CcpA* knockout *streptococcus bovis* S1 incubation with high glucose, LGWT: wild type *streptococcus bovis* S1 incubation with low glucose, LGKO: *CcpA* knockout *streptococcus bovis* S1 incubation with low glucose. ** *p* < 0.01, * *p* < 0.05, ns *p*>0.05, NS. means can’t be compared the two group for didn’t detect the metabolite. *CcpA*: Catabolite control protein A.


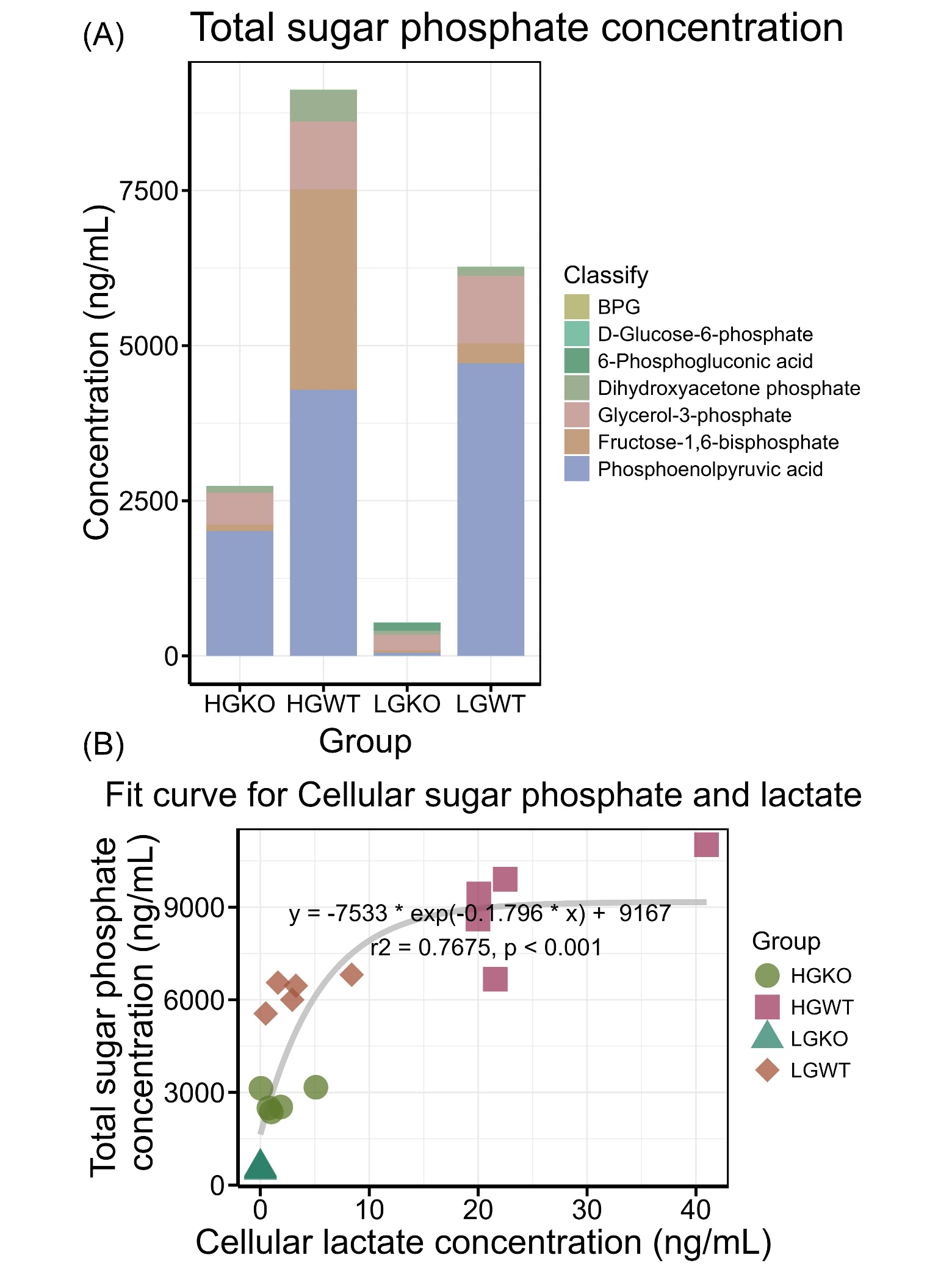


**Fig. S4(A).** Cellular total sugar phosphate concentration among HGWT, HGKO, LGWT, and LGKO groups. Fig. S4(B). Fit curve between cellular total sugar phosphate and cellular lactate. HGWT: *Streptococcus bovis* S1 wild type incubation with high concentration glucose, HGKO: *CcpA* knockout *streptococcus bovis* S1 incubation with high glucose, LGWT: wild type *streptococcus bovis* S1 incubation with low glucose, LGKO: *CcpA* knockout *streptococcus bovis* S1 incubation with low glucose.
